# Advanced Face Mask Filters Based on PCL Electrospun Meshes Dopped with Antimicrobial MgO and CuO Nanoparticles

**DOI:** 10.3390/polym14163329

**Published:** 2022-08-16

**Authors:** Carolina A. M. Ferreira, Sara F. C. Guerreiro, Joana F. A. Valente, Tatiana M. F. Patrício, Nuno Alves, Artur Mateus, Juliana R. Dias

**Affiliations:** 1 Centre for Rapid and Sustainable Product Development (CDRSP), Instituto Politécnico de Leiria, 2030-028 Marinha Grande, Portugal; 2 Abel Salazar Institute of Biomedical Sciences (ICBAS), University of Porto, Rua de Jorge Viterbo Ferreira, 228, 4050-313 Porto, Portugal; 3 Centro de Estudos de Ciência Animal (CECA), Instituto de Ciências, Tecnologias e Agroambiente (ICETA) da Universidade do Porto, Praça Gomes Teixeira, Apartado 55142, 4051-401 Porto, Portugal; 4 Marine and Environmental Sciences Centre (MARE), ESTM, Instituto Politécnico de Leiria, 2050-641 Peniche, Portugal; 5 Medical Physics Department, Portuguese Institute of Oncology (IPO-Porto), 4200-072 Porto, Portugal; 6 Instituto de Investigação e Inovação em Saúde (i3S), Universidade do Porto, Rua Alfredo Allen 208, 4200-135 Porto, Portugal

**Keywords:** advanced mask filter, antimicrobial nanofibers, electrospun meshes, copper oxide nanoparticles, magnesium oxide nanoparticles

## Abstract

The pandemic situation caused by coronavirus clearly demonstrated the need for alternatives able to protect the respiratory tract and inactivate the infectious agents. Based on this, antibacterial face-mask filters of polycaprolactone (PCL) dopped with magnesium oxide (MgO) and copper oxide (CuO) nanoparticles (NPs) were produced using an electrospinning technique. A morphological analysis of electrospun meshes evaluated the success of nanoparticles’ incorporation as well as the average fibers’ diameters (481 ± 272 nm). The performance of electrospun nanofibers was also assessed in terms of tensile strength (0.88 ± 0.25 MPa), water vapor permeability (11,178.66 ± 35.78 g·m^−2^·day^−1^), stability under wet conditions and antibacterial activity according to the standard guidelines. The filters showed structural stability up to 2 h of washing and improved antibacterial activity against *Escherichia coli* (*E. coli*) and *Staphylococcus aureus* (*S. aureus*) for optimized concentrations of MgO and CuO NPs. Overall, electrospun meshes with antibacterial activity were successfully developed for advanced filtering applications.

## 1. Introduction

In the past couple years, global health has focused on the coronavirus disease 2019 (COVID-19) pandemic, and consequently, the concern about respiratory-tract diseases caused by fine particulate matters (PMs), pathogenic aerosols and volatile organic compounds increases. Beyond COVID-19, the most common respiratory infectious diseases are influenza (cold), pneumonia, legionella, middle east respiratory syndrome (MERS), and severe acute respiratory syndrome (SARS), which are caused by virus, and pneumococcal meningitis and tuberculosis caused by bacteria [1,2].

The transmission of those pathogen agents is made in two different ways: contact or contact-free. In the first case, virus might be passed by direct contact (such as a handshake) or indirect contact, through the contamination of objects/surfaces (fomites) followed by direct contact with face mucosa (eyes, mouth or nose) [2]. Contact-free transmission occurs mainly by airway and is the most common method of contamination, by the virus’s attachment to human liquid particles, such as cough, sneezing, speech and breath of infected persons when in close unprotected contact [2,3]. These liquid particles can vary in size, ranging from respiratory droplets (>5 µm diameter) to smaller aerosols (<5 µm diameter) [4]. Nonetheless, respiratory droplets can remain present on surfaces, such as fomites, while the aerosols can be inhaled in crowded or inadequately ventilated spaces [3].

**Figure 1:**
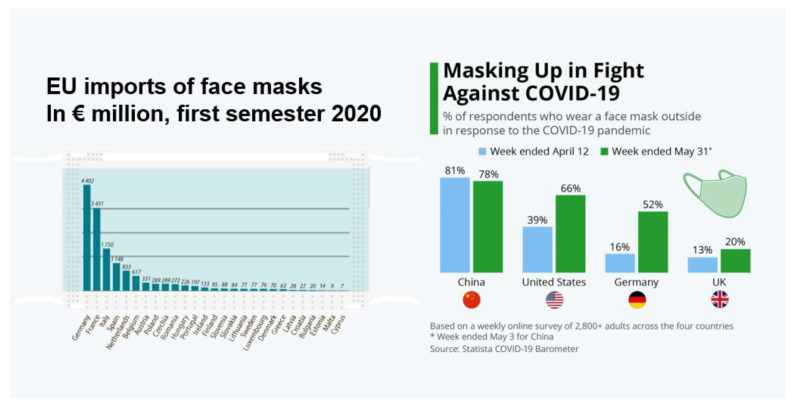
Differences among EU member states about face-mask importation costs (in EUR million) and % of people, by country, that use face masks to prevent COVID transmission. Source: EUROSTASTS and COVID-19 Barometer 2020.

Due to the high rate of transmission, the use of personal protective equipment (PPE) is crucial to stop the pathogenic agents spread and recommended by the World Health Organization (WHO) [5]. In fact, due to the COVID-19 pandemic, the use of PPE was unprecedented with a higher demand across the world, with the market growing 16.58% from 2019 to 2020. In addition, this market is expected to rise from EUR 79 billion in 2022 to EUR 108 billion by 2029, exhibiting a Compound Annual Growth Rate (CAGR) of 4.7%, according to the forecaster Fortune Business Insights [6]. Currently, the most commercialized PPE are surgical masks as well as some respirators and filters. Additionally, in EU comparing the first semester of 2020 with the same period of 2019, the importation of face masks grew dramatically, from EUR 800 million to EUR 14 billion, registering an increase of 1800% [7]. Comparing the member states, it is observed that countries with a higher population imported more face masks than smaller countries (Figure 1). Nevertheless, a survey data from countries such as the United States (U.S.), China, Denmark and United Kingdom (UK) reveals that people continue to wear face masks despite some pandemic restrictions being lifted [8]. Regarding the demanding consumption of those items, it may compromise the virus non-transmission since their manufacturing technique consists of thicker multilayers of spunbonded and melt-blown polypropylene (PP) [9]. In fact, the common PPE is composed of large diameter fibers from 500 to 1000 nm, leading to microscale pores and the common respiratory infectious agents (bacteria and virus) ranging in size of 20–500 nm, and indeed the average size of SARS-CoV-2 is 100 nm [4,10]. Additionally, PP masks only provide protection through fibers morphology and lose their capacity to maintain electrostatic charges over time, reducing even more their filtering efficiency [11,12].

**Figure 2:**
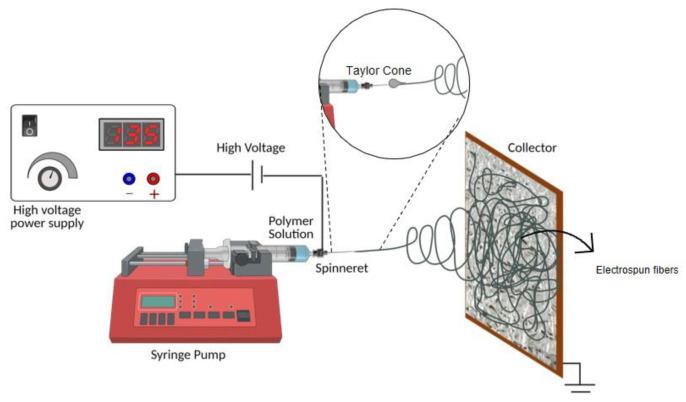
Schematic representation of electrospinning apparatus.

Electrospinning allows the production of nano (<1000 nm) to microfibers (>1 μm) based on high voltage that can overcome the surface tension of a polymeric solution to form fibrous structure through the complete evaporation of the solvent [12] (Figure 2). This technique possesses unique features such as an endless use of polymers, high surface-area-to-volume ratio, adaptable surface morphology, high porosity and interconnectivity and tailored mechanical performance, and it is possible to add bioactive particles, biomolecules and/or drugs, making electrospun meshes attractive for air filtration applications. Other advantages of this technique are the low-cost production versus the scale-up process, and production of a relatively high number of fibers in reduced time [12]. Currently, there are some studies available using electrospun nanofibers to prevent SARS-CoV-2 spreading, namely by Leung and Sun (2020), who produced poly(vinylidene fluoride) (PVDF) electrospun meshes with >90% of filtering capacity of nano-aerosols with 100 nm [13]. Polylactic acid (PLA) with activated charcoal membranes were developed by Bulus et al. (2020) to be used as face masks for the long-term, especially for healthcare professionals [14]. Another strategy to produce nanofiber masks and filters capable of reducing the spread of SARS-CoV-2 and inhibiting their activity is through the incorporation of antimicrobial agents [15,16]. These agents include metals, such as NPs of magnesium oxide (MgO), copper oxide (CuO), silver (Ag) and zinc (Zn), among others, which, due to their positive charge, can interact with the negatively charged cell wall of those pathogenic organisms [17], causing membrane disruption and damage of viral genome [4,18,19]. The use of metallic NPs in electrospun fibers was already reported successfully to combat other viruses such as influenza A [20], herpes, porcine parvovirus [21] or the bacteriophage MS2 [21,22], as well as bacteria such as *E. coli*, *Bacillus subtilis* (*B. subtilis*) and *Staphylococcus gallinarum* (*S. gallinarum*), demonstrating high anti-bacterial activity (>98% inhibition) [23,24,25].

Based on this, the main aim of this study was the production of PCL meshes containing MgO and CuO NPs to create advanced antimicrobial mask filters to combat the spread of pathogenic agents such as SARS-CoV-2. The polymer chosen to produce electrospun fibers was PCL, because it is easy to process and is biodegradable, biocompatible and nontoxic [26], and its hydrophobic nature promotes aerosol repulsion. Moreover, the final product certification might be facilitated when PCL is used in formulation since this polymer is FDA-approved for biomedical devices [27].

## 2. Materials and Methods

### 2.1. Materials

Polycaprolactone (PCL, Mw 50,000 (g·mol^−1^), bulk density: 1.1 g·cm^−3^) was kindly supplied by Perstorp (Malmo, Sweden). The acetone (ACE, 2.5 L, 67-64-1, ≥99.8%) used in the solution’s formulation was purchased from Sigma-Aldrich (St. Louis, MO, USA) with analytical grade and used without any further purification. MgO NPs (40–60 nm, 1309-48-4, ≥99.9%) and CuO NPs (II, 30–50 nm, 1317-38-0, ≥99.9%) were obtained from Alfa Aesar GmbH & Co KG, (Erlenbachweg, Kandel, Germany). Luria Bertini broth (L.B. Medium, 610084-6100845-620084) was acquired from Liofilchem^®^ (Via Scozia, Roseto degli Abruzzi, Italy). Luria Bertani Agar, Miller (Miller Luria Bertani Agar, M1151-500 G) was purchased from HiMedia^®^ (Laboratories Private Limited, Thane West, Maharashtra, India) and Tween^®^ 80 (polyethylene glycol sorbitan monolaurate) BioChemica (A1390) was obtained from PanReac AppliChem ITW Reagents (Castellar del Vallès, Barcelona, Spain).

### 2.2. Methods

#### 2.2.1. Electrospinning Meshes Preparation

Firstly, 16 wt% PCL, 10 wt% MgO NPs and 5 wt% CuO NPs were added to the vial simultaneous, according to the PCL mass, then acetone was used as a solvent. The solution was kept stirring overnight at 37 °C. After PCL dissolution, the solution was placed into the ultrasound bath (ArgoLab, DU-45, Carpi Mo, Italy) for 90 min at room temperature. Subsequently a sonication probe (UP200 Ht—Handheld Ultrasonic Homogenizer, 200 W, 26 kHz, Hielscher Ultrasonics GmbH, Oderstr, Teltow, Germany) was used according to the methodology defined previously [28]; briefly, during 40 min sonication cycles of on/off (45 s/10 s) were applied, with an amplitude of 80% and a power of 50 W to achieve full NPs dispersion. The electrospun meshes dopped with MgO/CuO NPs were obtained using an electrospinning homemade apparatus, applying the same processing parameters as the control condition (PCL meshes), namely: a high voltage of 15 kV, a flow rate of 0.8 mL·h^−1^ and 15 cm of distance from tip-to-collector. A single electrospun mesh production was completed after 20 min.

#### 2.2.2. Physico–Chemical Characterization

##### Apparent Density and Porosity

High porosity is crucial to guarantee the breathability and to provide high surface area. Based on this, apparent density and porosity of electrospun meshes using the Equations (2) and (3) were calculated:

(1) $${\mathrm{Apparent}\;\mathrm{density}\;(g\text{·}\mathrm{cm}}^{-3})=\;\frac{\mathrm{mesh}\;\mathrm{mass}\;(g)}{{\mathrm{mesh}\;\mathrm{thickness}\;(\mathrm{cm})\text{·}\mathrm{mesh}\;\mathrm{area}\;(\mathrm{cm}}^{2})}$$

(2) $$\mathrm{Mesh}\;\mathrm{porosity}\;\left(\%\right)=\frac{\mathrm{mesh}\;\mathrm{apparent}\;\mathrm{density}\;\left(g\cdot {\mathrm{cm}}^{-3}\right)}{\mathrm{Bulk}\;\mathrm{density}\;\mathrm{of}\;\mathrm{PCL}+\mathrm{MgO}\;\mathrm{NPs}+\mathrm{CuO}\;\mathrm{NPs}\;\left(g\cdot {\mathrm{cm}}^{-3}\right)}$$

##### Fourier Transform Infrared Spectroscopy with Attenuated Total Reflection

The functional chemical groups of the MgO and CuO NPs and electrospun meshes dopped with MgO/CuO NPs were determined by Fourier transform infrared spectroscopy with attenuated total reflection (FTIR-ATR, Alpha-P, Bruker, Ettlingen, Germany), in the range of 4000–400 cm^−1^, at a 4 cm^−1^ of resolution with 64 scans.

##### Scanning Electron Microscopy Analysis and Energy-Dispersive X-ray Spectroscopy

The morphology of electrospun meshes were evaluated by Scanning electron microscopy (SEM, TESCAN, Vega3-LMU, Kohoutovice, Czech Republic). Prior to the analysis, the meshes were coated with gold/palladium (Au/Pd) thin film by sputtering (Quorum Technologies, Lewes, UK). SEM images were also used to evaluate the fiber diameter distribution using Image J software (Fiji, version J1.46r., NIH, Bethesda, MD, USA), to each condition three individual samples were analyzed and fifty measurements per image were made. Through Energy-Dispersive X-ray (EDX, Brucker Spectroscopy, Xflash 6|30, Würzburg, Germany) an EDX-mapping of electrospun meshes was also achieved to acquire a better knowledge of how NPs are distributed into the fibers.

##### Water Vapor Permeability

The water permeation rate through electrospun meshes dopped with MgO/CuO was estimated and compared with PCL electrospun meshes and commercialized TNT surgical masks based on the ASTM E 96-00 standard test for water vapor transmission [29]. In addition, an evaluation was performed combining dopped meshes with commercial TNT to overcome the small thickness of electrospun meshes that can limit its usage. Five equal glass bottles were filled with 5 mL of phosphate-buffered saline (PBS) solution for each sample condition and meshes attached to the vial’s aperture with a vapor permeation area of 3.60 cm^2^.

To calculate the electrospun meshes’ water permeation rate, the vials were weighed before the assay and after 24 h of incubation at 32 °C, following the equation:

(3) $$\mathrm{Water}\;\mathrm{vapor}\;\mathrm{permeability}\;\left(\mathrm{WVP}\right)=\frac{\Delta W}{\mathrm{tA}}$$

where ∆W is water weight change (g), t is the time (h), and A is the test area (vials aperture area), in m^2^.

##### Nanoparticle’s Stability

The structural integrity of electrospun meshes is crucial for filtering applications to reduce the risk of metal oxides being released and reach the lungs [30]. Furthermore, previous studies [31,32] describe laboratory washing procedures to evaluate the potential migration of NPs from the membrane. In this context, the electrospun filters produced were submitted to a simulated washing procedure to evaluate their wash durability and potential release of metal oxide NPs. The potential amount of metal oxide NPs released was measured considering their concentration in the washing medium, as well as the weight loss by electrospun filters during the process. For this purpose, the membranes were weighed (Wi) using a microbalance (Sartorius, CPA 26P, Gottingen, Germany) and placed in glass test tubes filled with 15 mL of distilled water used as washing medium. Then, the glass tubes were placed in the incubator chamber (KS 4000, IKA, Staufen, Germany) under stirring for 2 h at 37 °C. After that, the samples were placed on a 96-well plate and analyzed in the UV/Vis spectrophotometer (BMG LABTECH, SPECTROstarNano, Ortenberg, Germany). The absorbance of these samples was read at the wavelength of 400 nm, which was reported in the literature for the Cu and Mg NPs [4,5]. Additionally, aqueous suspensions of CuO and MgO with known concentrations (C1, C2 and C3) were previously prepared and also analyzed in the UV/Vis spectrophotometer. These concentrations are shown in Table 1 and correspond to a different percentage of the total weight of NPs initially incorporated in the electrospun fibers, namely 100, 50 and 1%. Therefore, for a release of 1% of the total amount of CuO NPs from electrospun meshes, a concentration of 2.1 × 10^−4^ (g/mL) is expected in the washing medium.

At the same time, a second set of meshes were dried for 48 h in the incubator chamber, and their weight (Wf) was measured. The weight loss by electrospun filters was quantified by subtracting the final weight (Wf) from the initial weight (Wi) of the membrane. This process allows a double verification of the potential release of the particles in harsh conditions, both by UV/Vis spectrophotometry and weight loss.

#### 2.2.3. Antimicrobial Activity

The single and combined antibacterial activity of different concentrations (0, 1, 5, and 10 wt%) of MgO and CuO incorporated in PCL electrospun meshes was evaluated. The samples were cut in the dimension of simple square test pieces of 15 mm × 15 mm and irradiated under UV light for 15 min on each side to sterilize. Then, the antibacterial activity was assessed against the Gram-negative *E. coli* (ATCC 8739) and Gram-positive *S. aureus* (ATCC 6538P) through the ASTM E218007—standard test used for determining the activity for antimicrobial surfaces. Bacteria colonies of *E. coli* and *S. aureus* were resuspended in 0.85% (*w*/*v*) saline solution (NaCl) and turbidity adjusted to 0.5 McFarland (approximately 108 CFU·mL^−1^). To prepare the inoculated agar, 500 µL of bacterial inoculum was added to the 50 mL agar medium and agitated slowly. To evaluate the antibacterial activity, square mesh samples were cautiously daubed with 500 µL the inoculated agar slurries, which were then recovered from the samples after 24 h in contact during incubation at 37 °C. After incubation, the meshes with the inoculated agar slurries were added to a test tube with 5 mL of NaCl 0.85% (*w*/*v*) and Tween and homogenized carefully until detachment of inoculated agar slurries was observed. Subsequently, 100 µL of NaCl 0.85% (*w*/*v*) and Tween was added to a 1.5 mL tube with 900 µL NaCl 0.85%, and then sequential dilutions (9:1) were performed by the other four 1.5 mL tubes. This procedure was repeated for each condition (oxide concentration, bacteria). After that, viable bacteria were counted by the pour plate culture method [33], in which, 10 µL of the previous serial dilutions test tubes was deposited on the agar plate, divided by 3 drops and incubated at 37 °C for 24 h. All plating was performed in triplicate. After incubation, for each dilution series, bacterial reduction was calculated accordingly with the following equation:

(4) $$\mathrm{Bacterial}\;\mathrm{reduction}\;\left(\%\right)=\frac{C-B}{C}\;\times \;100$$

where B represents the number of CFUs obtained with the samples and C is the CFUs of bacteria obtained from positive control. To minimize error measures, the number of colonies in the Petri dish were accounted for if containing 30 to 300 colonies. After this initial screening, the same antibacterial activity protocol was applied to the PCL/MgO/CuO (16/10/5 wt%) electrospun meshes, although the contact between the electrospun meshes and the bacterial sludge was performed for determined time-points to mimic the maximum time recommended to use the face masks, namely 4 h, 6 h and 8 h.

#### 2.2.4. Mechanical Characterization

Mechanical characterization of electrospun meshes was determined by the tensile strength at break (TSB), Young’s modulus (YM) and Elongation at break (EB). Prior to performing the tensile tests, at least 5 measures of sample thickness were calculated. The tensile tests were performed in the texturometer TA.XT Plus model (Stable Micro System SMD, Surrey, UK) with a 5N load cell, a gauge length of 10 mm, and a test speed of 1 mm s^−1^ until failure. Ten individual samples were tested from each group and measurements were reported as mean ± standard deviation (SD).

#### 2.2.5. Statistical Analysis

All results were displayed as mean ± SD. In apparent density, porosity assessment, fiber diameter, and nanoparticle stability, to verify the normality of the data, a Kolmogorov–Smirnov test was used, and then a *t*-test was applied. In mechanical tests, normality was confirmed by the Shapiro–Wilk test, followed by a *t*-test in YM, TSB and EB. In the assessment of the antibacterial activity, the Shapiro–Wilk test was used to ensure the normality, and a one-way Analysis of Variance (ANOVA) followed by the Tukey test was performed. A *p*-value ≤ 0.05 was considered statistically significant. All the statistical analyses were performed using SigmaPlot (version 11.0, Systat Software Inc., San Jose, CA, USA) with a 95% confidence.

## 3. Results and Discussion

### 3.1. Physico–-Chemical Characterization

#### 3.1.1. Apparent Density and Porosity

The filter performance is mainly influenced by its structure; in this case, the electrospun meshes morphology is affected by fiber diameter, porosity and consequently apparent density, which can be correlated with the processing time. The obtained results for PCL electrospun meshes and meshes dopped with the MgO and CuO NPs can be observed in Table 2.

The introduction of NPs into PCL electrospun fibers presented a significant influence on the structure’s morphology, specifically in fiber diameter, porosity and apparent density (*p* ≤ 0.05). The introduction of such metallic NPs does not affect the meshes’ thickness since the processing parameters were maintained, such as the processing time.

The slightly reduction of fiber diameters can be correlated to the incorporation of MgO and CuO NP’s that increase the charges of the solution resulting in higher elongation forces, and consequently reducing the fiber diameter [32]. Additionally, higher fiber diameters lead to a bigger pore size as demonstrated in the literature [34]. Moreover, higher ultrasonication time decreases the solution’s viscosity, and consequently the electrical conductivity increases. This phenom is also explained by the energy caused by ultrasonic cavitation, which promotes the de-agglomeration of the MgO/CuO NPs, leading to a uniform NPs dispersion and an increasing of electrical conductivity [35].

#### 3.1.2. Fourier Transform Infrared Spectroscopy with Attenuated Total Reflection

**Figure 3:**
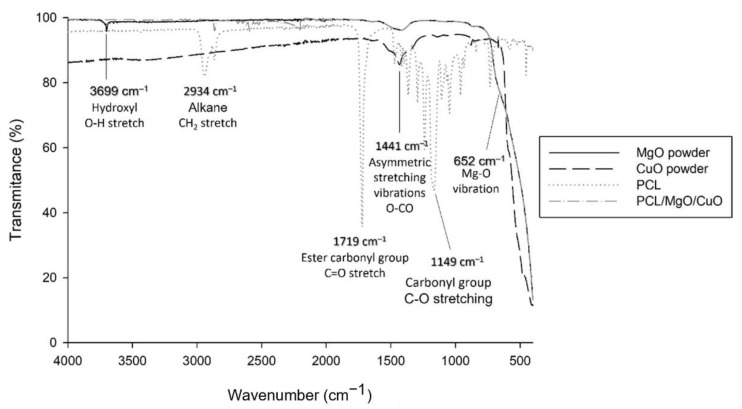
FTIR-ATR spectra of the MgO and CuO NPs powder and PCL electrospun meshes as controls, and PCL/MgO/CuO electrospun meshes with the main peaks identified and functional groups.

The FTIR-ATR was performed to demonstrate the successful integration of MgO and CuO NPs into the PCL electrospun fibers. According to the spectra obtained (Figure 3), the band observed in 2934 and 2852 cm^−1^ is attributed to CH_2_—stretch in PCL and PCL/MgO/CuO spectra. In 1719 cm^−1^ region at the PCL and PCL/MgO/CuO spectra the mentioned bands correspond to the -C = O (carbonyl stretch in ester carbonyl group from PCL polymer). The region that comprises the wavenumber of 1472, 1366, 1247, 1164 and 1149 cm^−1^ is ascribed to C-H scissoring, C-O stretching symmetric, asymmetric C–O–C stretching, symmetric C–O–C stretching and carbonyl group C-O stretching respectively, in PCL and PCL/MgO/CuO spectra [36,37,38].

In the commercial MgO powder spectrum, the bands visible in the region of 652 cm^−1^ until 400 cm^−1^, belong to the Mg-O vibration [39]. Additionally, the narrow band at the wavenumber ~1434 cm^−1^ might indicate the flexural vibration of the surface hydroxyl groups since MgO tends to absorb H_2_O and CO_2_ from the atmosphere [40]. The band at 3699 cm^−1^ reveals the presence of MgO nanoparticles [41].

Regarding the CuO NPs spectra, the main bands observed in the region of ~1441 cm^−1^ and in 879 cm^−1^ could be due to the presence of CO_2_ on the sample’s surface, forming carbonate structures (CuCO_3_) [42]. The characteristic absorption bands of CuO are sited in a region of 400–460 cm^−1^ consistent to the Cu–O stretching vibrations, visible on the CuO powder and PCL/MgO/CuO spectra [43].

#### 3.1.3. Scanning Electron Microscopy Analysis and Energy-Dispersive X-ray Spectroscopy

**Figure 4:**
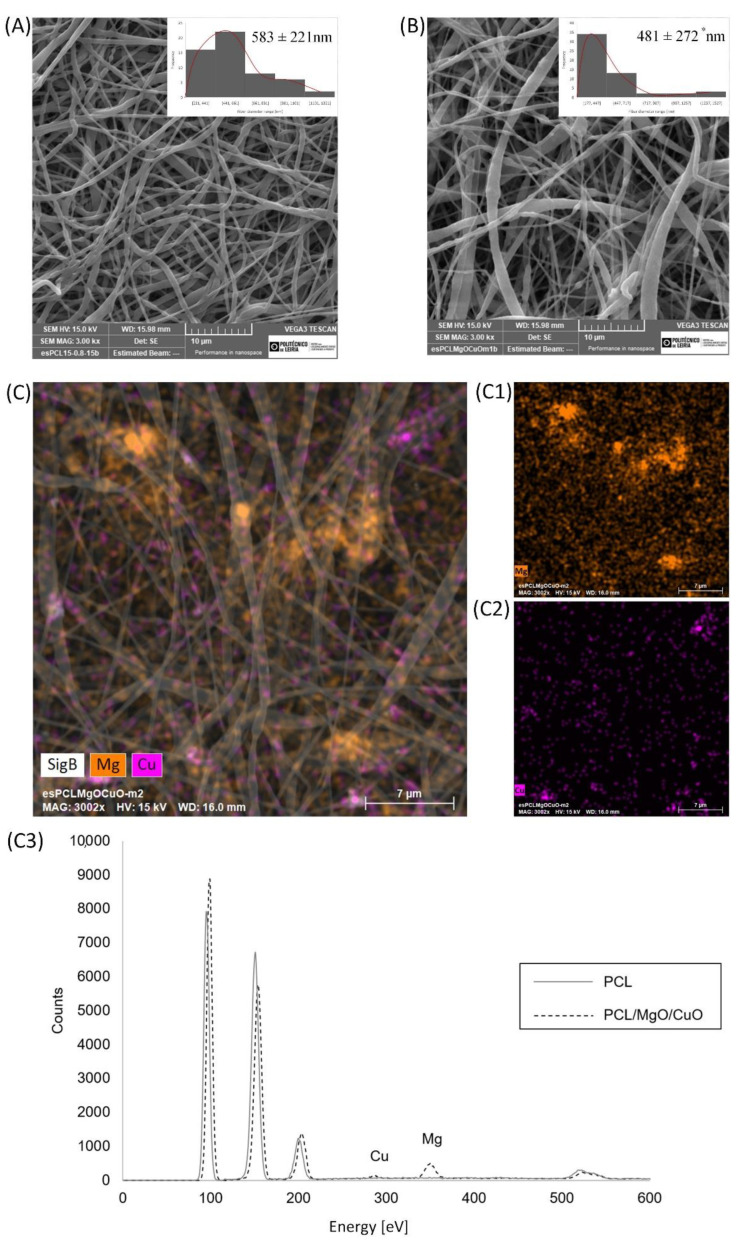
Scanning electron microscopies of PCL electrospun meshes and respective average fiber diameters with the distribution curve (**A**). PCL electrospun meshes dopped with MgO and CuO NPs with the average fiber diameters and its distribution curve (**B**). Statistical significance for *p* ≤ 0.05 (*). (**C**) EDX mapping combined with SEM image of PCL electrospun meshes dopped with MgO and CuO NPs (orange: MgO NP (**C1**); purple: CuO NP (**C2**)), (**C3**) EDX spectrum of PCL-free and non-free MgO and CuO NPs.

SEM images in Figure 4A,B, showed the morphology structure and average fiber distribution of the electrospun meshes developed. Overall, the fiber morphology presents a random distribution with no beads or drops. The PCL electrospun meshes present a fiber diameter of 583 ± 221 nm and 481 ± 272 nm for PCL/MgO/CuO, respectively. As mentioned previously, the introduction of the oxides reduces the fiber diameter as compared to the PCL (*p* > 0.05). Observing the SEM images, PCL fibers seem to be more homogenous, which is also verified with the distribution curves, while in the electrospun PCL/MgO/CuO meshes some beads remain, resulting from the total non-dispersion of the oxides, originating some variability during the jet stretching. This heterogeneity is also observed by the non-normal distribution on curves.

EDX was performed to evaluate the dispersion of CuO and MgO NPs into the fibers (Figure 4C) through the element mapping. According to the mapping obtained, it is possible to observe the NPs (CuO and MgO) integrated into the fibers as well as a homogeneous distribution of the NPs with limited aggregation. Combining the EDX and SEM results, it is possible to correlate the influence of NPs agglomeration with the beads formation.

#### 3.1.4. Water Vapor Permeability

**Figure 5:**
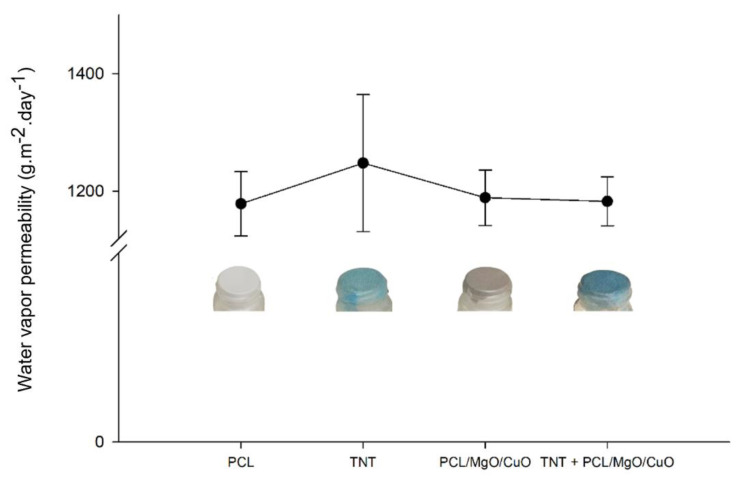
Water vapor permeability test for PCl electrospun meshes, commercial TNT, PCL electrospun meshes dopped with CuO/MgO NPs, and TNT attached to PCL/MgO/CuO electrospun mesh. No statistical significance was observed between samples (*p* > 0.05 (*n* = 5)).

Pore size is one of the most important factors for the filtering mask development, since it will influence the breathability, drop pressure and consequently particle absorption. As proof-of-concept of PCL electrospun meshes dopped with CuO and MgO NPs, the water vapor permeability (WVP) was tested and compared with the PCL samples, commercial TNT and commercial TNT combined with electrospun PCL/MgO/CuO meshes as presented in Figure 5.

According to Figure 5, it can be observed that PCL, PCL/MgO/CuO and TNT + PCL/MgO/CuO samples present similar values, such as 1167.97 ± 45.66, 1178.66 ± 35.78 and 1174.48 ± 33.98 g·m^−2^·day^−1^ without statistical differences (*p* > 0.05). The notable oscillations on commercial TNT sample values (1226.74 ± 101.06 g·m^−2^·day^−1^) can be related to: (i) the big pore-size consequence of the large fibers’ diameters (~24 µm), and (ii) to the morphology heterogeneity consequence of the common industrial process used. In fact, reducing the fiber diameters does not compromise the WVP of the structure, since the values are not statistically significant compared to the commercial TNT.

#### 3.1.5. Nanoparticle’s Stability

**Figure 6:**
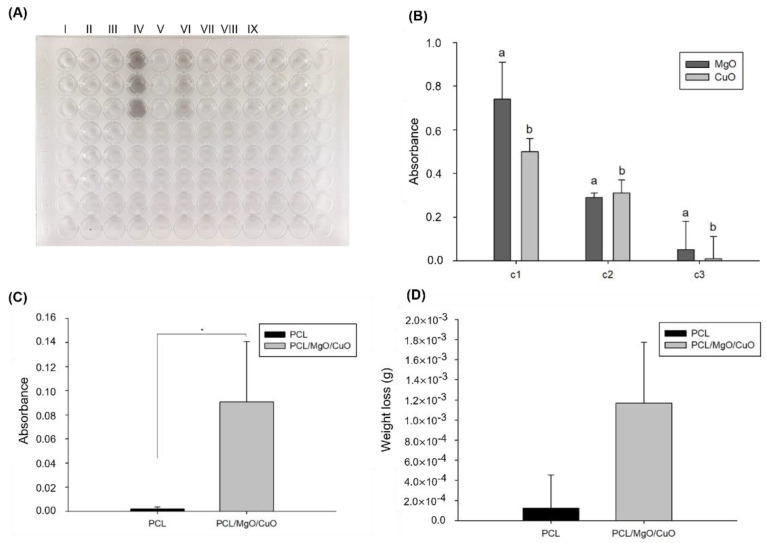
Stability test during washing process of electrospun filters. (**A**) 96-well plate used in the spectrophotometry assay for metal oxide NPs released quantification (I—control; II—PCL mesh; III—PCL/MgO/CuO; IV—CuO 100%; V—MgO 100%; VI—CuO 50%; VII—MgO 50%; VIII—CuO 1%; IX—MgO 1%); (**B**) Absorbance representing 100% (c1), 50% (c2), and 1% (c3) of the total amount of nanoparticles (measured at 400 nm); (**C**) Absorbance of the aqueous medium for 2 h of washing (measured at 400 nm); (**D**) Weight loss during washing process. Differences between conditions were evaluated all pairwise using Tukey’s test. Bars with the same letters have statistically significant differences (*p* > 0.05). Statistical significance for *p* ≤ 0.05 (*).

The stability of electrospun meshes as filters was evaluated in terms of the amount of metal oxide NPs released during the washing procedure. The results showed that CuO and MgO NPs when dispersed in aqueous media create dark and white opaque solutions, respectively. As observed in Figure 6A, these differences are only visually noticeable for high concentrations of metal oxide NPs (>50% of total nanoparticles released). Therefore, the spectroscopic analysis of these solutions was used to quantify the differences through their absorbance values. Both CuO and MgO NPs exhibit significant differences (*p*-value ≤ 0.05) between the absorbance values for the three concentrations considered (c_1_, c_2_ and c_3_) as shown in Figure 6B. As expected, absorbance is directly proportional to the concentration of nanoparticles. However, the method only showed sensitivity for concentrations above 1% since during preliminary tests no significant differences could be identified between the absorbance values obtained for different concentrations under this value.

Furthermore, samples of washing medium previously stored for 2 h of stirring were also analyzed using spectroscopy. In this situation, it was possible to identify significant differences (*p*-value ≤ 0.05) between the washing medium after contact with electrospun filters without NPs (control) and the washing medium in contact with electrospun filters dopped with NPs. In addition, after 2 h of washing and stirring, the absorbance values obtained for the washing medium were inferior to the values obtained for 1% of CuO and MgO released. These results suggest a potential vestigial release of metal oxide NPs inferior to 1% of the total weight of nanoparticles initially incorporated (Figure 6C).

Since UV-Vis spectrophotometry does not have sensitivity to detect differences between low concentrations, a complementary strategy was considered to evaluate the nanoparticle’s stability based on the weight loss by the electrospun filters after washing. The results obtained did not show significant differences between the control and electrospun filters containing NPs, demonstrating that there is no increased risk of release compared to the control situation as shown in Figure 6D. In fact, the mean values of weight loss are consistent with the maximum concentration determined by spectrophotometry. Additionally, the high heterogeneity of the samples also contributes to an increase in the error in the results. Future improved methodologies should consider samples of electrospun membranes in the same range of weight as an additional quality control filter to avoid error contributions due to structural heterogeneity.

Following that, the security of the membrane is ensured for up to 2 h of use equivalent to continuous washing. The impact of humid and turbulent environments in the filters developed was quantified; however, it is expected that this period highly increases for normal and correct daily usage. These results suggest that the electrospun meshes dopped with oxides have strong stability during the washing process and consequently present an advantage compared to the control.

### 3.2. Antimicrobial Activity

**Figure 7:**
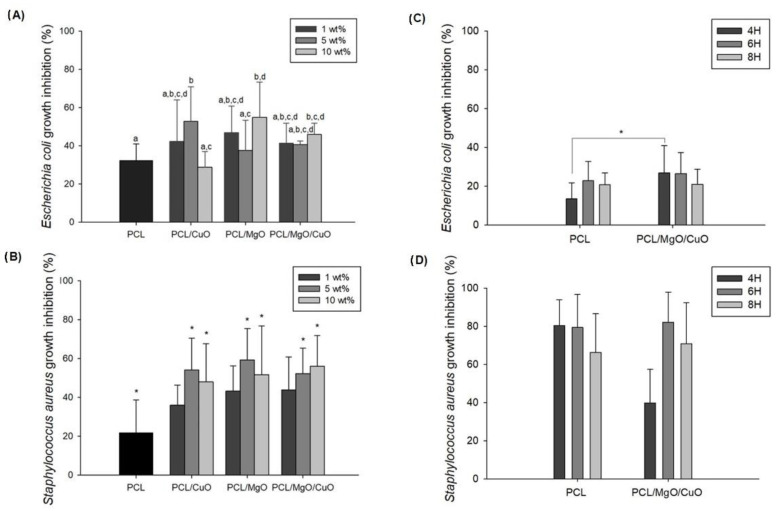
Antibacterial activity of PCL electrospun meshes dopped with different concentrations of CuO NPs (1, 5 and 10 wt%) and MgO NPs (1, 5 and 10 wt%), as well as electrospun meshes with equal incorporation (1:1) of CuO and MgO NPs tested against *E. coli* (**A**) and *S. aureus* (**B**), compared with the control condition (PCL electrospun meshes). *E. coli* (**C**) and *S. aureus* (**D**) growth inhibition at 4 h, 6 h and 8 h after direct contact with electrospun mesh dopped with the final formulation (5 wt% CuO/10 wt% of MgO NPs) and PCL electrospun mesh as a control. For each assay, the differences between conditions were evaluated all pairwise using Tukey’s test. Bars with the same letters do not have statistically significant differences (*p* > 0.05). Statistical significance for *p* ≤ 0.05 (*).

To mitigate health risks caused by some pathogenic agents (e.g., virus, bacteria and fungus) antimicrobial electrospun meshes can be used as a bioactive filter of face masks. The antimicrobial activity of electrospun meshes dopped with MgO and CuO NPs was evaluated using two different bacterial strains: *E. coli* (ATCC 8739) as Gram-negative and *S. aureus* (ATCC 6538P) as Gram-positive (Figure 7A,B). Firstly, we evaluated PCL electrospun meshes dopped with different concentrations of MgO and CuO NPs (1, 5, and 10 wt%) and that combination to understand which condition presents a higher antimicrobial effect. Results demonstrated that 10 wt% of MgO and 5 wt% of CuO were the conditions with higher antimicrobial action. After that we assessed the antimicrobial activity of 16 wt% of PCL electrospun meshes dopped with 10 wt% of MgO and 5 wt% CuO by direct contact for different time-points (4 h, 6 h and 8 h).

As observed in Figure 7A, the addition of 5 wt% of CuO and 10 wt% of MgO to PCL electrospun meshes improves the antibacterial activity against *E. coli* (*p* ≤ 0.05). Combining the CuO with the MgO the condition with better performance was the one with 10 wt% (1:1) of NPs compared to the PCL samples (*p* ≤ 0.05). PCL electrospun meshes, used as control, presented an inhibition of *E. coli’s* growth in 32.14 ± 8.82%, while in CuO samples, the highest concentration inhibited 52.78 ± 18.05%, and in MgO samples, the maximum activity recorded was 54.89 ± 18.50%. The narrow antibacterial activity observed in the control condition can be associated to the structure morphology (small pore size) and to the polymer hydrophobicity that reduce the bacterial infiltration [29].

As observed in Figure 7B, several formulations of PCL electrospun meshes dopped with metal oxides showed antibacterial activity against *S. aureus*. The antibacterial activity was observed for both metal oxides (CuO and MgO NPs), with the concentrations of 5 and 10 wt%. Similar to the results obtained with *E. coli*, antibacterial behavior of *S. aureus* was detected for single and combined NPs. However, the higher antibacterial activity was identified for PCL electrospun meshes dopped with 5 wt% of MgO presenting an inhibition rate of 59.20 ± 16.30%, although without significant differences. These results are supported by previous studies [44,45,46] reporting the antibacterial efficacy of MgO and CuO NPs against *S. aureus*. According to Phan et al., the improved antibacterial activity with CuO against *S. aureus* when compared with *E. coli* can be the result of structural differences in the bacterium cell that promote the attachment and the interaction between the cell membrane and the NP [46]. Another study conducted by Liu et al. reported the higher efficacy of MgO NPs against *E. coli* compared to *S. aureus,* and even in the present work the results do not suggest such behavior. In fact, the ability of NPs to inactivate pathogens depends on several factors such as small size, high specific surface area, surface charge to cross the pathogens membrane, binding properties to host cells, and particle concentration, retention, bioavailability and stability [4,47]. According to the results obtained with *S. aureus* assay, Figure 7B, the intermediate concentrations exhibited slightly higher antibacterial activity compared with higher concentrations that might be correlated with the NPs aggregation, since available surface area is reduced as well as their availability and, consequently, their antimicrobial activity [47].

Additionality, the antibacterial activity of the electrospun meshes dopped with CuO NPs can be explained by the particle size, since the CuO NPs in this study had a dimension around 30–50 nm, being inferior to that of MgO nanoparticles, which reveal greater contact surface area and higher dispersion rate.

Overall, MgO presented higher antimicrobial activity than CuO NPs since MgO NPs have a higher action spectrum on Gram-negatives, such as *E.* *coli* [44,48]. The mechanism of how MgO induces cellular death is not fully understood, but according to Maji et al., the antibacterial activity is caused by the great amount of oxygen moieties present in the MgO surface that are adsorbed on the bacteria cell wall, forming reactive oxygen species (ROS) [38]. The ROS will disrupt the carbonyl group of the peptide’s bonds in cell wall and the membrane, leading to the leakage of intracellular materials followed by cell death. Another study suggests that the MgO antibacterial mechanism is attributed to the lipid peroxidation induced by hydrogen peroxide (H_2_O_2_) [49].

In Figure 7C, the antibacterial activity against *E. coli* was not linear over time. In fact, after 4 h of contact, bacterial growth inhibition was 26.94 ± 13.96% and after 6 h the inhibitory activity decreased from 26.50 ± 10.81 to 20.03 ± 7.80%. Comparing the PCL and PCL/MgO/CuO electrospun meshes over the time, the inhibitory activity was noticed only for 4 h of contact (*p* ≤ 0.05). The same trend was observed for PCL electrospun meshes coated with silver nanoparticles (AgNPs) that showed excellent antibacterial action against *E. coli* in 4 h, suggesting that after 4 h some growth mechanism is disrupted in this strain, inhibiting the bacteria activity [50].

Similarly, in Figure 7D, it is possible to observe variation in the inhibition rate against *S. aureus* over time. The antibacterial activity of PCL/MgO/CuO electrospun meshes was evaluated for time-points of 4, 6 and 8 h. At the initial exposure of 4 h, the PCL membranes showed a higher rate of inhibition (80.4 ± 13.5%) of bacterial growth compared to the solution, including the NPs (39.7 ± 17.7%) that might be associated to the structure morphology. The addition of CuO and MgO NPs to the electrospun meshes demonstrated the increase of antimicrobial activity over time. Moreover, dopped electrospun meshes show their greatest effectiveness in the 6 h (82.1 ± 15.08%) and 8 h (70.8 ± 21.5%) of contact, the maximum time recommended to use the face mask.

It is worth noticing that the antimicrobial activity can also be explained based on the air filter mechanism, which in this case is electrostatic filtration since due to the nanoparticle’s charges, the pathogenic particles (bacteria, viruses) are attracted to the electrospun surface, interacting trough intermolecular interactions, namely formation of ROS. Regarding the time-point assay, the overall results revealed less antimicrobial activity of PCL electrospun meshes dopped with MgO/CuO NPs against *E. coli* compared to *S. aureus* bacteria, resulting from the morphological differences on cell wall, namely due to the greater number of proteins (peptidoglycan) in the cell membrane and an additional outer layer of *E. coli* compared to *S. aureus*.

### 3.3. Mechanical Characterization

**Figure 8:**
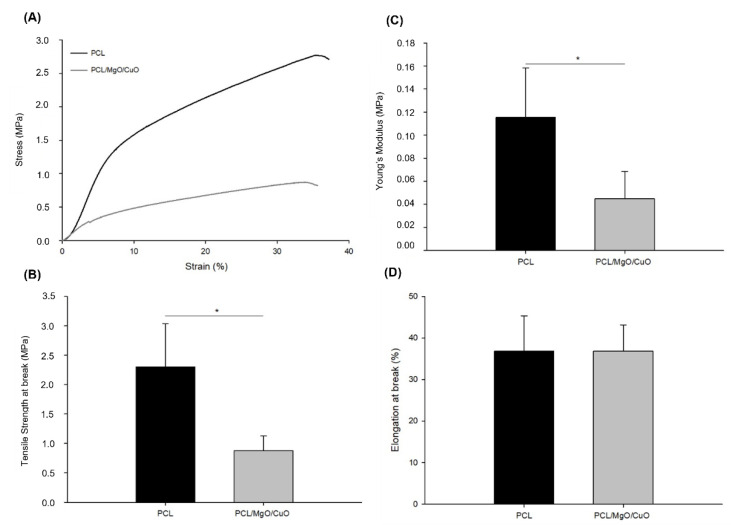
Mechanical characterization of PCL electrospun meshes (black) and PCL electrospun meshes dopped with MgO and CuO NPs (grey). (**A**) stress–strain representative curves; (**B**) Young’s Modulus; (**C**) tensile strength at break; and (**D**) elongation at break. Statistical significance for *p* ≤ 0.05 (*).

Uniaxial tensile tests were performed to evaluate the mechanical properties of PCL electrospun meshes and PCL dopped with CuO and MgO NPs. In Figure 8), it is possible to observe the representative stress–strain curves for both conditions of PCL and PCL/MgO/CuO electrospun meshes. Figure 8A–D represents Young’s Modulus (YM), Tensile Strength at break (TSB) and Elongation at break (EB), respectively.

According to the results obtained, the dopped structures present lower YM (0.045 ± 0.02 MPa) than PCL electrospun meshes without NPs (0.115 ± 0.04 MPa). Since YM is inversely proportional to the structure elasticity, and considering previous studies reported in literature [51,52], we expected a decrease of YM and an increase of tensile strength after the incorporation of metal oxide NPs in electrospun nanofibers. In the present study, the incorporation of small concentrations of metal oxides NPs in polymeric nanofibers contribute to significant morphological changes in fiber diameters and a structural arrangement of nanofibers that show impact in final mechanical properties of electrospun nanofibers. Furthermore, the concentration of particles incorporated was not enough to observe an increase of the stiffness of the structure; on the other hand, the changes caused during production led to an elastic behavior of the meshes produced.

In regard to the TSB, it is also higher in PCL samples, with 2.30 ± 0.73, and 0.88 ± 0.25 MPa in PCL/MgO/CuO, with statistical differences (*p* ≤ 0.05) supporting the distinctive morphological structures, such as average fiber diameter and porosity as result of the introduction of MgO and CuO NPs. According to previous literature [23], those results were not expected since the addition of metallic nanoparticles improve tensile strength on nanofibers meshes. The appearance of some remaining beads at the structure, as visualized before on Figure 4, can also contribute for such behavior. The beads exhibited a larger average diameter compared to the fibers, being an obstacle to pass the load, eventually breaking. Other studies corroborate the influence that dispersion of the nanoparticles had in the improvement of the mechanical properties, especially Young’s modulus and tensile strength. In fact, in another work, we previously tested the influence of two different methods of CuO/MgO nanoparticles dispersion on the properties of electrospun meshes [28]. Only the EB displays similar values, 36.80 ± 8.49 and 36.79 ± 6.37% for PCL and PCL/MgO/CuO, respectively (*p* > 0.05).

Overall, it was expected that with decreasing fiber diameter, as in PCL/MgO/CuO, the mechanical properties of the electrospun fiber increase [51]. This effect is related to the crystallinity of thinner fibers, which is higher than the crystallinity of thicker fibers. Consequently, the higher degree of molecular orientation along the thinner-fibers’ axis under high extensional flow during electrospinning is observed, which leads to a drastic change in the elastic modulus [52,53]. However, despite the fiber diameter of PCL/CuO/MgO being smaller and the porosity, compared with the PCL samples, the mechanical behavior was lower, reflecting a ductile performance.

## 4. Conclusions

Electrospun meshes of PCL dopped with CuO/MgO NPs were successfully produced and its antibacterial activity was evaluated against two different bacteria strains, *E. coli* and *S. aureus*. The optimal concentration of metal oxides proved its efficiency against both strains for up to 24 h of contact. The results suggested that antibacterial activity of encapsulated CuO/MgO is observed without the release of nanoparticles, and the structural integrity of electrospun membranes and nanoparticles’ stability were maintained after washing and stirring for up to 2 h. Furthermore, morphological analysis and mechanical tests proved the influence of metal oxides in fiber stretching and heterogeneity. However, the mechanical, morphological and functional changes observed in the performance of PCL meshes when dopped with MgO/CuO NPs proved to be consistent for filtering applications. The desired electrospun nanofibers offer filtering capacity and additional antibacterial activity when compared with current solutions commercially available. In fact, to our knowledge, no studies are reporting the use of combined MgO and CuO for the development of protective respiratory filters. This work is the first study based on the synergetic effect of MgO and CuO NPs for filtering applications. The efficient use of the developed electrospun membranes for advanced and functional face masks with improved antibacterial activity can contribute to reducing public health problems associated with respiratory infections.

## Figures and Tables

**Table 1:** Concentrations of metal oxide NPs (CuO and MgO) used in spectroscopic analysis during washing test.

Type of NP		% of NPs Incorporated	Concentration of NPs (g/mL)
CuO	C1	100	2.1 × 10^−2^
	C2	50	1.1 × 10^−2^
	C3	1	2.1 × 10^−4^
MgO	C1	100	4.3 × 10^−2^
	C2	50	2.1 × 10^−2^
	C3	100	2.1 × 10^−2^

**Table 2:** Apparent density and porosity of the PCL electrospun meshes, and PCL electrospun meshes dopped with MgO and CuO NPs (PCL/MgO/CuO).

	Mesh Thickness (mm)	Apparent Density (g·cm^−3^)	Porosity (%)	Average Fibber Diameter (nm)
PCL	0.035 ± 0.002	0.23 ± 0.04	93 ± 1.19	583 ± 221
PCL/MgO/CuO	0.037 ± 0.011	±0.05 *	92 ± 1.47 *	481 ± 272 *

* Represent statistical differences between PCL/MgO/CuO and the control condition (*p* ≤ 0.05).

## Data Availability

The raw data required to reproduce these findings cannot be shared at this time as the data also form part of an ongoing study.
